# Supplementary material for: The GEN-ERA toolbox: unified and reproducible workflows for research in microbial genomics
Source: Gigascience. 2023 Apr 10;12:giad022. doi: 10.1093/gigascience/giad022 (PMC10084500; doi:10.1093/gigascience/giad022)
Supplement: giad022_Response_to_Reviewer_Comments_Original_Submission [file giad022_response_to_reviewer_comments_original_submission.pdf]

Author's Response To Reviewer Comments

Close

GIGA-D-22-00292

The GEN-ERA toolbox: unified and reproducible workflows for research in microbial genomics
Luc Cornet; Benoit Durieu; Frederik Baert; Elizabet D'hooge; David Colignon; Loic Meunier; Valérian Lupo; Ilse Cleenwerck; Heide-Marie Daniel; Leen Rigouts; Damien Sirjacobs; Stéphane Declerck; Peter Vandamme; Annick Wilmotte; Denis Baurain; Pierre Becker
GigaScience

Dear Dr Cornet,

Your manuscript "The GEN-ERA toolbox: unified and reproducible workflows for research in microbial genomics" (GIGA-D-22-00292) has been assessed by our reviewers. Based on these reports, and my own assessment as Editor, I am pleased to inform you that it is potentially acceptable for publication in GigaScience, once you have carried out some essential revisions suggested by our reviewers.

Their reports, together with any other comments, are below. Please also take a moment to check our website at https://www.editorialmanager.com/giga/ for any additional comments that were saved as attachments.

We have a few Editorial issues that need to be addressed at the same time. Please make sure the code has an OSI-approved open license and this should be listed in the code section of the paper.

> The GEN-ERA toolbox has a GPL-3.0 license, which is now mentioned in the section “Availability of supporting source code and requirements”

Line 490 of the tracked change document.

In addition, please register any new software application in the bio.tools and SciCrunch.org databases to receive RRID (Research Resource Identification Initiative ID) and biotoolsID identifiers, and include these in your manuscript.

> The biotools unique ID (biotools:gen-era_toolbox) and the RRID of SciCrunch.org (SCR_023114) have been added to the section “Availability of supporting source code and requirements”.

Line 492 of the tracked change document.

It's also important that computational workflows should be registered in workflowhub.eu and the DOIs cited in the relevant places in the manuscript. These will facilitate tracking, reproducibility and re-use of your tool.

> The workflows have been registered in workflow.eu (https://workflowhub.eu/workflows/416) and this is now mentioned in the manuscript, in the section “Availability of supporting source code and requirements”.

Line 493 of the tracked change document.

One other issue is Singularity has been recently renamed as Apptainer, so it would make sense to state that in the introduction.

> In the Introduction, we added that Singularity is now renamed Apptainer, but we decided to keep the

name Singularity for the rest of the paper since the tool is still named Singularity from the Apptainer website (https://apptainer.org/admin-docs/master/installation.html).

Line 88 of the tracked change document.

Once you have made the necessary corrections, please submit a revised manuscript online at:

https://www.editorialmanager.com/giga/

If you have forgotten your username or password please use the "Send Login Details" link to get your login information. For security reasons, your password will be reset.

Please include a point-by-point within the 'Response to Reviewers' box in the submission system. Please ensure you describe additional experiments that were carried out and include a detailed rebuttal of any criticisms or requested revisions that you disagreed with. Please also ensure that your revised manuscript conforms to the journal style, which can be found in the Instructions for Authors on the journal homepage. If the data and code has been modified in the revision process please be sure to update the public versions of this too.

The due date for submitting the revised version of your article is 02 Apr 2023.

We look forward to receiving your revised manuscript soon.

Best wishes,

Hongling Zhou
GigaScience
www.gigasciencejournal.com

We thank the reviewers for their helpful suggestions. We did our best to address the comments. Please find below our point-by-point response to the reviewers.

REVIEWER 1

Reviewer reports:
Reviewer #1: Paper Title: The GEN-ERA toolbox: unified and reproducible workflows for research in microbial genomics
The GEN-ERA toolbox provides a number of containerized workflows to researchers (without any specific training in bioinformatics) to study the diversity of well-characterized strains for fundamental and applied research. More specifically It facilitates all steps from genome downloading and quality assessment, including genomic contamination estimation, to tree phylogenetic reconstruction. It additionally provides workflows for average nucleotide identity comparisons and metabolic modeling.
The supplementary file provides details of how to run the whole workflow (through 10 steps), found in the GEN-ERA toolbox on basal, for an empirical dataset of early emerging cyanobacteria. It provides an up-to-date phylogenomic analysis of the Gloeobacterales order, the first group to diverge in the evolutionary tree of Cyanobacteria.
The github repo located at https://github.com/Lcornet/GENERA also provides more details about the GEN-ERA tools suite. Though in the manuscript it is mentioned that the call to Mantis could not be included in the Singularity call, on the github repo they have indicated that Mantis is now installed in a singularity container for the Metabolic workflow (install is no longer necessary).

> The problem with Mantis was the accession to the database outside the singularity container, it was impossible to connect Mantis within a container. During the review process, we took the decision to install the database inside the container (available from the DOX page), making the installation of Mantis by users not necessary anymore, and resolving the connection issue. This has been changed in the manuscript.

Line 202 of the tracked change document.

The tool has been tested on an empirical dataset of 18 (meta)genomes of early-branching Cyanobacteria and the time taken as well as the results of the run are documented in the supplementary file.
The authors claim that the tools suite can be used to study the diversity of microorganisms, including bacteria and fungi. From the github repo, it is clear that a number of publications in high-impact journal papers have already resulted from the development of the GEN-ERA.

1) Are the methods appropriate to the aims of the study, are they well described, and are necessary controls included?
This study aims at describing a toolbox, named GEN-ERA, and the methods section defines the various steps of the tools suite. Looking at the supplementary file and the github, it is easy to follow the manuscript. The versions of the programs used in the case study are provided in the forms of nextflow scripts.

> Thank you.

2) Are the conclusions adequately supported by the data shown?
The results of running the tools suite on an empirical dataset of 18 (meta)genomes of early-branching Cyanobacteria, at each step, as well as the time taken to download the files and the running each step, are convincing that it works fine, at least for Cyanobateria.
But this is found in the Supplementary Material. There should be section on Discussion and Conclusion in the main text.

> Thank you for this suggestion. A paragraph at the end of the case study has been added to emphasize and summarize the usage of the GEN-ERA toolbox. The format of the paper, technical note, did not authorize a conclusion within the main text. In consequence, we choose to add this paragraph at the end of the finding section.

Line 276-294 of the tracked change document.

3) Please indicate the quality of language in the manuscript. Does it require a heavy editing for language and clarity? But t
The use of English language is adequate and concise and can be understood clearly, by researchers interested in studying diversity of micro-organisms.

4) Are you able to assess all statistics in the manuscript, including the appropriateness of statistical tests used?

The statistics involved in the phylogenetic analyses are integrated in the existing programs. Hence I am not able to assess the statistics.

> Noteworthy, we did not design new statistical analyses, but report analyses provided by the programs used by the toolbox.

5) Final Comments
The proposed toolbox/toolsuite described in this manuscript is very relevant and worth a read for researchers interested in studying the diversity of microorganisms, including bacteria and fungi, especially as it helps to facilitate their life through the use of well-defined containerized NextFlow workflows.

> Thank you

I strongly believe that there should be a section on the Discussion of the results of running the toolbox for the case study and a Conclusion in the main manuscript. This will help readers in understanding the importance of the toolbox better.

> A paragraph has been added at the end of the finding section, see previous comment.

Line 276-294 of the tracked change document.

REVIEWER 2

Reviewer #2: Cornet et al have generated a collection of NextFlow pipelines which provide a pipeline to analyse data associated with genome or raw sequencing data of microbial organisms and protists. The methodology appears sound and reproducible. My main concern with the manuscript is that it is not well described in the abstract, introduction or GitHub repository. It isn't clear whether the analyses are specific for genomics questions arising from culture collections, or if it is more broadly applicable. There is also no discussion about other pipelines which achieve similar things e.g. ATLAS https://metagenome-atlas.github.io/

> A paragraph at the end of the case study has been added to emphasize and summarize the usage of the GEN-ERA toolbox. A sentence has been added to emphasize that our toolbox is designed for comparative genomics of both bacteria and small eukaryotes, which was not the purpose of other pipelines. Although the toolbox was developed for culture collection, it can be used on any genomic data, as it is showed by our case study. A sentence on this subject has also been added into this paragraph.

Line 276-294 of the tracked change document.

I also had a number of minor concerns, detailed below.

A number of grammatical errors detected, these should be fixed. Parts of the manuscript are also slightly too informal e.g. "This confirms the interest of 221using ORPER to spot interesting SSU rRNA sequences"

> The sentence has been deleted.

It would be helpful if the GitHub front page could provide a concise description of what the software aims to achieve, to make its use more understandable.

> A description of the toolbox has been added at the GitHub front page.

“The GEN-ERA toolbox is a suite of Nextflow-Singularity workflows designed for comparative genomics of bacteria and small eukaryotes. Without any installation, it allows researchers to download, assemble and bin (meta)genomes (from short or long reads). Orthologous inference and maximum likelihood phylogenomic analyses (bootstrap and jackknife) can be inferred with this suite. Constrained (by a ribosomal phylogenomic) SSU rRNA phylogeny can also be inferred. Average nucleotide identity, GTDB identification and metabolic modelling are also included in the toolbox.”

106: "as it happened" grammatical error

> The sentence has been modified

"Assembly.nf" Commonly assembly is a separate process to binning, but here binning has been included. Perhaps a clearer name might be Genome-recovery.nf ?

> The workflow was originally created for genome assembly only and then we had more option like the binning. It was more useful for users to have all these topics into one single workflow. Nevertheless, we did not choose to change the names of the tool because it was widely used under its former name of Assembly.nf. It is indeed used on several ongoing project/papers and it would be difficult to change now.

124: "Researchers interested in a better understanding of these tools can read the recent review on the

detection of genomic contamination made by Cornet et al. [15]." While not inappropriate, this is perhaps too much self-citation.

> The sentence has been deleted

Why is contamination assessed but not completeness?

> The completeness is also estimated, it has been added in the manuscript.

Line 131 and 143 of the tracked change document.

129: "annotation of bacterial proteins is automatic" Automatic in what sense? Annotation also refers to describing the function of the protein usually, but here the meaning appears to be restricted to ORF calling. I found this somewhat confusing. Also "in the different GEN-ERA workflows" is unclear - does this mean that prodigal is run as part of the Assembly.nf workflow for instance?

> The annotation here means prediction of proteins. We have now specified this in the manuscript. We also added the names of the workflows where the bacterial protein prediction is included.

Line 146-148 of the tracked change document.

143: "Orthology.nf automatically provides the core genes, shared by all the organisms in unicopy" what is meant by "all organisms" here?

> It is user dependent. This can be all the organisms provided to orthology.nf or the user can choose to exclude the outgroup for instance. We added new options concerning this to this workflow and explained them in the wiki. In the text, we replaced "all organisms" by "all genomes provided by the user".

Line 161 of the tracked change document.

145: "The OGs of proteins 145 can be further enriched" what does "enriched" mean?

> By "enriched", we mean to add orthologous sequence to an OG, without having to run a new orthologous inference. It is now specified in the manuscript.

Line 164 of the tracked change document.

163: GTDB.nf is described in the "Other workflows" section, when it is phylogeny-related.

> We only use GTDB in the toolbox to classify genomes, which is more taxonomy related. This is why it is treated in "Other workflows". It is now specified in the manuscript.

Line 195 of the tracked change document.

172: "it was 173 technically not possible to include Mantis in a container" I am curious as to why this was the case? I do not have any specific insight or ability to judge the accuracy of this statement, just curious. Inclusion of a sentence describing the difficulties might help other workflow developers and/or the Mantis developers.

The problem with Mantis was the accession to the database outside the singularity container, it was impossible to connect Mantis within a container. During the review process, we took the decision to install the database inside the container (available from the DOX page), making the installation of Mantis by users not necessary anymore, and resolving the connection issue. This has been changed in the manuscript.

Line 202 of the tracked change document.

190: "Gloeobacterales are the most basal order of the 191 Cyanobacteria phylum" This statement is somewhat controversial, because the GTDB has defined the Melainobacteria as being a part of the Cyanobacteria phylum based on RED values. I would suggest removing "the most basal" or making it clear that cyanobacteria refers to photosynthetic cyanobacteria rather than the phylum.

> Indeed, this can be controversial. We now specify photosynthetic cyanobacteria.

Line 225 of the tracked change document.

189: The methods for this section are not described in the methods section. They are only briefly described in the Findings section. A clearer link to these methods should be made from the maintext and methods.

> A new section has been added to the methods to describe the case study.

Line 462-484 of the tracked change document.

212: Showed -> show.

> Done

215: "estimate the sequencing level of the order" it isn't clear what meaning this has.

> It is the presence and localization of genomes among the SSU rRNA diversity. We add this definition in the manuscript.

Line 251 of the tracked change document.

224: Our results demonstrate the absence of one metabolic 225pathway" There are many metabolic pathways, presumably it is missing more than one.

> Thank you, changed

233: "examples of the practical usage of the GEN-ERA toolbox are available in Supplemental 234File 1." this does not make it clear that this refers to the methods for this specific example.

> A method section has been added for the case study.

Line 462-484 of the tracked change document.

Close
