# Supplementary material for: The GEN-ERA toolbox: unified and reproducible workflows for research in microbial genomics
Source: Gigascience. 2023 Apr 10;12:giad022. doi: 10.1093/gigascience/giad022 (PMC10084500; doi:10.1093/gigascience/giad022)
Supplement: giad022_Reviewer_1_Report_Original_Submission — Shakuntala Baichoo -- 12/5/2022 Reviewed [file giad022_reviewer_1_report_original_submission.pdf]

Reviewer Report

Title: The GEN-ERA toolbox: unified and reproducible workflows for research in microbial genomics

Version: Original Submission **Date:** 12/5/2022

Reviewer name: Shakuntala Baichoo

Reviewer Comments to Author:

Paper Title: The GEN-ERA toolbox: unified and reproducible workflows for research in microbial genomicsThe GEN-ERA toolbox provides a number of containerized workflows to researchers (without any specific training in bioinformatics) to study the diversity of well-characterized strains for fundamental and applied research. More specifically It facilitates all steps from genome downloading and quality assessment, including genomic contamination estimation, to tree phylogenetic reconstruction. It additionally provides workflows for average nucleotide identity comparisons and metabolic modeling.The supplementary file provides details of how to run the whole workflow (through 10 steps), found in the GEN-ERA toolbox on basal, for an empirical dataset of early emerging cyanobacteria. It provides an up-to-date phylogenomic analysis of the Gloeobacteralesorder, the first group to diverge in the evolutionary tree of Cyanobacteria.The github repo located at https://github.com/Lcornet/GENERA also provides more details about the GEN-ERA toolssuite. Though in the manuscript it is mentioned that the call to Mantis could not be included in the Singularity call, on the github repo they have indicated that Mantis is now installed in a singularity container for the Metabolic workflow (install is no longer necessary).The tool has been tested on an empirical dataset of 18 (meta)genomes of early-branching Cyanobacteria and the time taken as well as the results of the run are documented in the supplementary file.The authors claim that the toolsuite can be used to study the diversity of microorganisms, including bacteria and fungi. From the github repo, it is clear that a number of publications in high-impact journal papers have already resulted from the development of the GEN-ERA.1) Are the methods appropriate to the aims of the study, are they well described, and are necessary controls included?This study aims at describing a toolbox, named GEN-ERA, and the methods section defines the various steps of the toolsuite. Looking at the supplementary file and the github, it is easy to follow the manuscript. The versions of the programs used in the case study are provided in the forms of nextflow scripts.2) Are the conclusions adequately supported by the data shown?The results of running the toolsuite on an empirical dataset of 18 (meta)genomes of early-branching Cyanobacteria, at each step, as well as the time taken to download the files and the running each step, are convincing that it works fine, at least for Cyanobateria.But this is found in the Supplementary Material. There should be section on Discussion and Conclusion in the main text.3) Please indicate the quality of language in the manuscript. Does it require a heavy editing for language and clarity? But tThe use of English language is adequate and concise and can be understood clearly, by researchers interested in studying diversity of micro-organisms.4) Are you able to assess all statistics in the manuscript, including the appropriateness of statistical tests used?The statistics involved in the phylogenetic analyses are integrated in the existing programs. Hence I am not able to assess the statistics.5) Final CommentsThe proposed toolbox/toolsuite described in this manuscript is very relevant and worth a read for researchers interested in studying the

diversity of microorganisms, including bacteria and fungi, especially as it helps to facilitate their life through the use of well-defined containerized NextFlow workflows.I strongly believe that there should be a section on the Discussion of the results of running the toolbox for the case study and a Conclusion in the main manuscript. This will help readers in understanding the importance of the toolbox better.

Level of Interest

Please indicate how interesting you found the manuscript: Choose an item.

Quality of Written English

Please indicate the quality of language in the manuscript: Choose an item.

Declaration of Competing Interests

Please complete a declaration of competing interests, considering the following questions:

- Have you in the past five years received reimbursements, fees, funding, or salary from an organisation that may in any way gain or lose financially from the publication of this manuscript, either now or in the future?
- Do you hold any stocks or shares in an organisation that may in any way gain or lose financially from the publication of this manuscript, either now or in the future?
- Do you hold or are you currently applying for any patents relating to the content of the manuscript?
- Have you received reimbursements, fees, funding, or salary from an organization that holds or has applied for patents relating to the content of the manuscript?
- Do you have any other financial competing interests?
- Do you have any non-financial competing interests in relation to this paper?

If you can answer no to all of the above, write 'I declare that I have no competing interests' below. If your reply is yes to any, please give details below.

'I declare that I have no competing interests'

I agree to the open peer review policy of the journal. I understand that my name will be included on my report to the authors and, if the manuscript is accepted for publication, my named report including any attachments I upload will be posted on the website along with the authors' responses. I agree for my report to be made available under an Open Access Creative Commons CC-BY license (http://creativecommons.org/licenses/by/4.0/). I understand that any comments which I do not wish to be included in my named report can be included as confidential comments to the editors, which will not be published.

Choose an item.

To further support our reviewers, we have joined with Publons, where you can gain additional credit to further highlight your hard work (see: https://publons.com/journal/530/gigascience). On publication of this paper, your review will be automatically added to Publons, you can then choose whether or not to claim your Publons credit. I understand this statement.

Yes Choose an item.
