# Supplementary material for: The GEN-ERA toolbox: unified and reproducible workflows for research in microbial genomics
Source: Gigascience. 2023 Apr 10;12:giad022. doi: 10.1093/gigascience/giad022 (PMC10084500; doi:10.1093/gigascience/giad022)
Supplement: giad022_Reviewer_2_Report_Original_Submission — Ben Woodcroft -- 1/2/2023 Reviewed [file giad022_reviewer_2_report_original_submission.pdf]

Reviewer Report

Title: The GEN-ERA toolbox: unified and reproducible workflows for research in microbial genomics

Version: Original Submission **Date:** 1/2/2023

Reviewer name: Ben Woodcroft

Reviewer Comments to Author:

Cornet et al have generated a collection of NextFlow pipelines which provide a pipeline to analyse data associated with genome or raw sequencing data of microbial organisms and protists. The methodology appears sound and reproducible. My main concern with the manuscript is that it is not well described in the abstract, introduction or GitHub repository. It isn't clear whether the analyses are specific for genomics questions arising from culture collections, or if it is more broadly applicable. There is also no discussion about other pipelines which achieve similar things e.g. ATLAS https://metagenome-atlas.github.io/I also had a number of minor concerns, detailed below.A number of grammatical errors detected, these should be fixed. Parts of the manuscript are also slightly too informal e.g. "This confirms the interest of 221using ORPER to spot interesting SSU rRNA sequences"It would be helpful if the GitHub front page could provide a concise description of what the software aims to achieve, to make its use more understandable.106: "as it happened" grammatical error"Assembly.nf" Commonly assembly is a separate process to binning, but here binning has been included. Perhaps a clearer name might be Genome-recovery.nf ?124: "Researchers interested in a better understanding of these tools can read the recent review on the detection of genomic contamination made by Cornet et al. [15]." While not inappropriate, this is perhaps too much self-citation.Why is contamination assessed but not completeness?129: "annotation of bacterial proteins is automatic" Automatic in what sense? Annotation also refers to describing the function of the protein usually, but here the meaning appears to be restricted to ORF calling. I found this somewhat confusing. Also "in the different GEN-ERA workflows" is unclear - does this mean that prodigal is run as part of the Assembly.nf workflow for instance?143: "Orthology.nf automatically provides the core genes, shared by all the organisms in unicopy" what is meant by "all organisms" here?145: "The OGs of proteins 145 can be further enriched" what does "enriched" mean?163: GTDB.nf is described in the "Other workflows" section, when it is phylogeny-related.172: "it was 173 technically not possible to include Mantis in a container" I am curious as to why this was the case? I do not have any specific insight or ability to judge the accuracy of this statement, just curious. Inclusion of a sentence describing the difficulties might help other workflow developers and/or the Mantis developers.190: "Gloeobacterales are the most basal order of the 191 Cyanobacteria phylum" This statement is somewhat controversial, because the GTDB has defined the Melainobacteria as being a part of the Cyanobacteria phylum based on RED values. I would suggest removing "the most basal" or making it clear that cyanobacteria refers to photosynthetic cyanobacteria rather than the phylum.189: The methods for this section are not described in the methods section. They are only briefly described in the Findings section. A clearer link to these methods should be made from the maintext and methods.212: Showed -> show.215: "estimate the sequencing level of the order" it isn't clear what meaning this has.224: Our results demonstrate the absence of one metabolic 225pathway"

There are many metabolic pathways, presumably it is missing more than one.233: "examples of the practical usage of the GEN-ERA toolbox are available in Supplemental 234File 1." this does not make it clear that this refers to the methods for this specific example.

Level of Interest

Please indicate how interesting you found the manuscript: Choose an item.

Quality of Written English

Please indicate the quality of language in the manuscript: Choose an item.

Declaration of Competing Interests

Please complete a declaration of competing interests, considering the following questions:

- Have you in the past five years received reimbursements, fees, funding, or salary from an organisation that may in any way gain or lose financially from the publication of this manuscript, either now or in the future?
- Do you hold any stocks or shares in an organisation that may in any way gain or lose financially from the publication of this manuscript, either now or in the future?
- Do you hold or are you currently applying for any patents relating to the content of the manuscript?
- Have you received reimbursements, fees, funding, or salary from an organization that holds or has applied for patents relating to the content of the manuscript?
- Do you have any other financial competing interests?
- Do you have any non-financial competing interests in relation to this paper?

If you can answer no to all of the above, write 'I declare that I have no competing interests' below. If your reply is yes to any, please give details below.

I declare that I have no competing interests

I agree to the open peer review policy of the journal. I understand that my name will be included on my report to the authors and, if the manuscript is accepted for publication, my named report including any attachments I upload will be posted on the website along with the authors' responses. I agree for my report to be made available under an Open Access Creative Commons CC-BY license (http://creativecommons.org/licenses/by/4.0/). I understand that any comments which I do not wish to be included in my named report can be included as confidential comments to the editors, which will not be published.

Choose an item.

To further support our reviewers, we have joined with Publons, where you can gain additional credit to further highlight your hard work (see: https://publons.com/journal/530/gigascience). On publication of this paper, your review will be automatically added to Publons, you can then choose whether or not to claim your Publons credit. I understand this statement.

Yes Choose an item.
